# Sexting at an Early Age: Patterns and Poor Health‐Related Consequences of Pressured Sexting in Middle and High School*

**DOI:** 10.1111/josh.13258

**Published:** 2022-10-17

**Authors:** Katalin Parti, Cheryl E. Sanders, Elizabeth K. Englander

**Affiliations:** ^1^ Department of Sociology Virginia Tech 225 Stanger St (0137) Blacksburg VA 24061; ^2^ Department of Psychological Sciences Metropolitan State University of Denver P.O. Box 173362, Campus Box 54 Denver CO 80217‐3362; ^3^ Massachusetts Aggression Reduction Center Bridgewater State University Burrill Office Complex (95 Burrill Avenue) Bridgewater MA 02324

**Keywords:** sexting, pressure, early‐onset, adolescent, middle school, high school

## Abstract

**BACKGROUND:**

Sexting is sending, receiving, or forwarding sexually explicit messages, images, or videos through electronic means. Research has examined sexting in high school and college students. This study seeks to add to the existing literature by exploring the nature of pressured or problematic sexting in middle school‐aged subjects.

**METHODS:**

We asked participants in public colleges in Massachusetts, Colorado, and Virginia, to recall their sexting‐related experiences in middle and high school. We utilized an online survey tool for data collection. We performed bivariate quantitative statistical analyses to examine attitudinal and behavioral differences, as well as motivations and consequences of adolescent sexting.

**RESULTS:**

The study revealed unique patterns of early‐onset sexting compared to sexting in later adolescence. Early‐onset adolescents typically start sexting before they become sexually active and are at a higher risk for poor outcomes associated with sexting, they are more likely to seek therapy. Early sexting is significantly more pressured than sexting in later adolescence.

**CONCLUSIONS:**

The study is an important contribution to the existing research on pressured sexting. Exploring pressured sexting at very early ages finds that early sexting activity is more likely pressured, creates more stress than later in life, and hence, it needs attention from school mental health professionals and education programs. The authors suggest that comprehensive sex education, including sexting should begin earlier than middle school to prevent risky online sexual behavior and provide for learning coping mechanisms for adolescents.

Sexting, defined as the sending, receiving, or forwarding sexually explicit messages, images, or videos through electronic means, has become an increasingly prevalent behavior among adolescents.^1^ Several international studies have demonstrated the wide prevalence of sexting behavior among adolescents and young adults, suggesting that the phenomenon has been steadily increasing in recent years.^2^, ^3^, ^4^ These studies have examined sexting in high school students. However, other researchers have noted that sexting among middle school students seems to be associated with worse outcomes. For example, in 2014, Rice et al's^5^ study of 1285 middle school students found that 5% had sent a sext and that sexting was associated with engaging in a wide range of sexual activities in early teens in correlation with high rates of sexually transmitted infections and teen pregnancies.^5^ Another study echoed those findings.^6^ A longitudinal study found that sexting among young adolescents was associated with poorer mental health outcomes.^7^

## Age groups differences in sexting

According to researchers, sexting is more common among high school students than middle school students, and asking another person to send nudes or semi‐nudes is more common among boys than girls.^8^ Older adolescents have received and forwarded more sexts than younger ones,^9^, ^10^ with no significant gender differences. In a meta‐analysis of 39 studies, Madigan and colleagues^11^ found that the prevalence of sexting increases with the age of participants (higher in sexually active cohorts than sexually inactive ones), year of data collection (prevalence of sexting increased over time in different cohorts), and sexting method (higher prevalence on mobile devices compared with computers). Consequently, as adolescents get access to smartphones at earlier ages, the onset of sexting behavior is expected to shift to earlier ages as well.

## Predictors/motivations of sexting

Sexting has been described as a natural consequence of and part of the process of sexual maturation.^12^ Therefore, why seek to reduce sexting? Although sexting can be a harmless sign of sexual maturation, it is potentially risky behavior and a gateway to the victimization of sextortion (the threatened dissemination of explicit, intimate, or embarrassing images of a sexual nature without consent, for the purpose of procuring additional images, sexual acts, money, or something else),^13^ online grooming, or cyberbullying.^14^ Teenagers represent a vulnerable group due to their limited self‐regulation, high susceptibility to peer pressure, and technophilia (defined as enthusiasm or obsession toward advanced technologies).^15^ Studies find various predictors for an increased likelihood of non‐consensual dissemination of sexting: being sexually active is a primary predictor.^16^, ^17^ Further predictors (motivations to sext) are the normalization of sexting^18^ and stronger positive attitudes toward disseminating sexts as being funny.^16^, ^17^ A high level of impulsivity and being male are further predictors of engaging in sexting behaviors.^17^

## Problematic sexting

Since research in sexting started in 2009,^19^ most studies have investigated why people engage in sexting. However, fewer studies have concentrated on problematic sexting behavior and its consequences. In one study, Van Ouytsel et al's^20^ adolescent (15 to 18‐year‐old) focus group participants mentioned 3 main ways in which sexting images could be abused: (1) they could be used to coerce or blackmail the victim, (2) they could be distributed out of revenge after the breakup of a romantic relationship, or (3) they could be forwarded or shown to peers in order to boast about having received the digital image. All forms of image abuse could be problematic.

## Gender differences in sexting and pressured sexting

Generally, more males and older adolescents receive or forward sexts, compared to females and younger adolescents.^9^ In a study about sexting in dating relationships of adolescents, Reed et al^21^ found that, although both girls and boys reported sexting behaviors, girls were more likely to report receiving pressure to sext and negative emotional responses to sending requests from a dating partner. Sexting in dating relationships involved different emotions for girls and boys. For girls, being older, among other factors, predicted more positive emotional reactions to sexting requests from a partner. In contrast, being younger predicted more negative emotional reactions for girls to sexting requests.

Women generally feel more pressured and threatened than men to sext.^20^, ^22^ In subsequent studies about adolescents' perceptions of sexting, Van Ouytsel and colleagues^20^, ^23^ found that girls feel more pressured to share intimate images in teen relationships due to fear of losing their boyfriends. Young adult women experienced more pressure to engage in sexting than young men. Pressure can manifest in many forms, such as asking for photos repeatedly, and women feel more pressured to respond to preserve their relationships.^24^, ^25^, ^26^, ^27^ Ross et al^28^ examined sexting coercion as part of intimate partner abuse and found that women were more likely than men to be coerced into sexting. Both sexting coercion and sexual coercion were significantly and independently related to negative mental health symptoms and sexual problems; sexting coercion was found to be a cumulative risk factor for nearly all of these adverse effects.

## Consequences of non‐consensual sexting

Mitchell et al^29^ revealed that 21% of teens appearing in or creating sexually explicit images and 25% of teens who received such images were feeling very or extremely upset, embarrassed, or afraid as a result. In Livingstone and Görzig's^30^ research, 24% of European 11 to 16‐year‐olds who reported they received sexually explicit images said that the image made them uncomfortable, upset, or feel they should not have seen it. Younger, female, and less sensation‐seeking participants and those with pre‐existing psychological difficulties were more likely to experience such harm.

Studies identified associations between sexting and adverse psychosocial outcomes such as anxiety, depression, and suicidal ideation.^14^, ^31^, ^32^ However, Van Ouytsel et al^23^ warned that context matters and associated only coerced or pressured sexting, with negative psychosocial outcomes.^33^, ^34^, ^35^, ^36^ For example, in a recent study on German 13 to 16‐year‐old adolescents, Wachs et al^37^ reported that non‐consensual and pressured sexting were positively related to depressive symptoms and non‐suicidal self‐harm, whereas consensual sexting was unrelated to these outcomes. Furthermore, the study revealed that boys engaged in more non‐consensual sexting than girls, whereas girls were more pressured to send sexts than boys. As per the negative consequences, girls, non‐minority adolescents, and non‐sexual minority adolescents experienced greater depressive symptoms and non‐suicidal self‐harm when they felt pressure to sext.^37^

The above literature suggests that sexting in middle school may be an inherently high‐risk activity, but many questions remain unanswered. Van Ouytsel's review of adolescent sexting pointed out that more research is needed on young teens to identify risky behaviors.^24^ For example, while it seems clear that not all sexters experience negative outcomes, but it is less clear which types of *young* teens are at highest risk.^19^ Therefore, this study seeks to add to the existing literature by exploring the nature of pressured or problematic sexting compared in early (middle school) and late adolescence (high school). Therefore, we examine the attitudes toward sexting and possible undesired or negative consequences of pressured sexting at early ages on a sample of 999, 18 to 23 years old adolescents with a retrospective cross‐sectional survey design. Furthermore, although the prevalence and patterns of deliberate and pressured sexting in adolescents have been researched, differences between early‐onset (middle school) and late adolescence (high school) sexting have not been comparatively studied in one sample. Most studies specifically examining peer pressure as a motivation to sext have been conducted on high school samples.^38^, ^39^, ^40^ Developmentally speaking, it is reasonable to assume that pressure and sexting may be dynamically different between, for example, 13‐ and 16‐year‐olds. Therefore, this paper intends to address this void by investigating patterns and possible negative consequences of early‐onset pressured sexting (ie, pressured or coerced sexting performed in middle school), in comparison to pressured or coerced sexting starting in high school. Furthermore, we hypothesize that early‐onset sexting behavior is significantly more coerced or pressured than sexting in later adolescence (in high school) since later adolescence sexting is mainly related to intimate relationships. Therefore, this exploratory study operates with 3 research questions:

RQ1: What are the patterns of early sexting compared to sexting in later adolescence?

RQ2: Is early sexting (in middle school) more pressured than later sexting (in high school)?

RQ3: Are early‐onset sexters (those who sexted first in middle school) at a higher risk of poor outcomes than later adolescent sexters (who sexted first in high school) when sexting?

## METHODS

### Procedures

We recruited survey participants from 3 large public universities in the United States: Bridgewater State University (MA), Metropolitan State University of Denver (CO), and Virginia Tech (VA) during the academic year of 2020/2021. Undergraduate students were eligible to participate in the 1‐time online survey, either for course credit or for entering a drawing for a gift card. Participation was voluntary and confidential. For those who were offered course credits, an alternative assignment was provided to ensure voluntariness. The research was approved by all 3 participating universities' Institutional Review Boards, ensuring voluntariness and anonymity.

### Participants

Altogether, 1066 students participated in the survey, 494 from Virginia, 327 from Colorado, and 245 from Massachusetts in a cross‐sectional survey design. Participation rate reflects the size of the colleges, Virginia Tech having the most undergrad students (30,016; 1.7% participation of the undergraduate student body), followed by the Metropolitan State University of Denver (19,194; 1.7% participation) and Bridgewater State University (9463; 2.6% participation).^41^ Because we wanted to concentrate on undergraduate college students born between 2000 and 2003, we excluded those younger than 18 and older than 23 years of age (n = 67) at the time of the survey participation. (Although students older than 23 still merit study, we focused on participants who could still easily recall relatively recent sexting experiences.) As a result, the sample consisted of 999 participants (328 males; 649 females; 22 non‐binary) in total from the 3 universities. The mean age of the sample was 20.48 years (SD = 1.26). Therefore, in this current analysis, we concentrated on their data.

### Instrumentation

The survey included demographic questions, retrospective questions about sexting experiences in middle and high school, and feelings about sexting (ie, was it pressured or deliberate). Studies show sensitive questions, including deviant behavior, are most likely answered when asked retrospectively.^42^, ^43^, ^44^, ^45^ The authors did not opt for using a certain scale explicitly because this survey was meant to be exploratory in nature, looking to expand general understanding of adolescent sexting. Furthermore, to our knowledge, there was no validated sexting scale available for adolescent participants at the time of the fielding. However, we used a survey instrument that has been piloted and utilized^19^, ^46^, ^47^ on a large student body in one of the participating colleges, and has been adjusted based on participation feedback.

### Data analysis

Prior to data analysis, Levene's test for homogeneity of variance was conducted on applicable data. Results revealed no significant differences in equality of variances. Data were analyzed using SPSS Statistics for Windows, Version 27.0. IBM Corp. Statistical analyses included *t* tests and chi‐square tests of independence.

## RESULTS

Most participants were females (64.9%), who were more likely (52.6%) than males (42.1%) to have engaged in sexting; however, non‐binary individuals sexted more (54.5%) than females. Most participants sexted first at the ages of 16 and 17, with early age onset distribution being slightly tilted toward females who were more likely to have started sexting before the age of 15 (19.9%) and at the age of 15 (21.4%) than males (before age 15:12.6%; at age 15: 9.9%) and non‐binary (before age 15: 11.1%; at age 15: 11.1%) individuals. In all 3 gender groups, participants were most likely to have sent sexts to the person they were in a relationship with (females: 35.1%; males: 26.2%; non‐binary: 22.2%). However, sexting was a way to attract or establishing casual physical intimacy outside of a committed relationship (“hook up”) with persons even outside of relationships for females (37.6%), non‐binary individuals (33.3%), and males (11.3%). Pressured sexting was prevalent in both females (any pressure at females: 77.5%) and non‐binary individuals (any kind of pressure on non‐binary people: 77.8%). Males, however, were most likely to have (68.4%) sexted free of pressure. Aligned with this result, most females (89.6%), non‐binary individuals (72.2%), and males (75.4%) agreed with the statement, “too many people are pressured to sext.” While most females (57.3%) agreed with the statement “females feel more expectations to sext,” fewer males (32.6%) and non‐binary individuals (27.8%) did so. All the above variables showed statistically significant relations (Table 1).

**Table 1:** Sexting Behaviors and Perceptions Based on Gender (Percentages)

Variable	Males (n = 328)	Females (n = 649)	Non‐Binary (n = 22)	Chi‐Square Tests of Independence (Comparing Males vs Females)
Ever engaged in sexting				
Yes	42.1	52.6	54.5	*χ* ^2^ (1) = 16.01
No	57.9	44.4	45.5	*p <* .001
				ϕ= 0.13*** n = 977
Age of 1st sext sent				
Before age 15	12.6	19.9	11.1	*χ* ^2^ (3) = 14.1
Age 15	9.9	21.4	11.1	*p* = .003
Ages 16‐17	42.3	35.9	66.7	V = 0.18**
Ages18 or older	35.1	22.8	11.1	n = 448
Who sext was sent to				
In relationship with person	26.2	35.1	22.2	*χ* ^2^ (5) = 21.62
Wanted to attract them	4.6	12.5	11.1	*p* = .001
Just wanted to hook up with them	6.7	2.9	22.2	*V* = 0.22**
A friend	2.6	4.4	33.3	n = 456
An adult known online	0.9	1.5	11.1	
Other	5.3	2.3	0.0	
Type of pressure felt to sext				
No pressure	68.4	22.5	22.2	*χ* ^2^ (4) = 93.82
Self‐pressure	7.0	18.1	22.2	*p* < .001
Friendly pressure	22.8	26.0	0.0	V = 0.45***
Pestered	0.9	25.4	25.4	n = 456
Very negative pressure	0.9	7.9	11.1	
Too many people are pressured to sext				
Yes	75.4	89.6	72.2	*χ* ^2^ (1) = 31.15
No	24.6	10.4	27.8	*p <* .001
				ϕ = 0.19*** n = 903
Females feel more expectations to sext				
Yes	32.6	57.3	27.8	*χ* ^2^ (1) = 47.41
No	67.4	42.7	72.2	*p <* .001 ϕ = 0.23*** n = 903

*p < .05. **p < .01. ***p < .001.

In the next step, we examined sexting‐related attitudes of those who had sexted (n = 427; 42.7%). While most participants sent their first sext during high school, slightly more females (19.9%) started sexting at middle school compared to males (12.6%) and non‐binary people (11.1%) (n.s.). Most participants, regardless of whether they sent their first sext during middle (MS) or high school (HS), thought that “sexting is fun and exciting” (MS: 68.3%; HS: 68.3%; n.s.), “sexting is safer than sex” (MS: 58.5%; HS: 56.3%; n.s.), “sexting means you like the person” (MS: 50.0%; HS: 58.7%; n.s.), and “sexting should only happen between people in established relationships” (MS: 50.0%; HS: 58.7%; n.s.). (Although it is worth mentioning that participants answered these questions when they were college students; hence, these reflect current opinion as opposed to their thinking as middle or high school teenagers.) The only statistically significant difference between middle and high school onset sexters was sexting's relation to a romantic relationship: while middle school sexters mostly (87.8%) sexted before any sexual activity, sexting was much more likely to be a follow up to actual sex for high school students (40.3%) (V = 0.29; *p* < .001) (Table 2).

**Table 2:** Sexting as Normative Behavior: Early‐Onset (Middle School; MS) vs Later Adolescence (High School; HS) Sexters

Variable	MS (n = 82)	HS (n = 375)	Chi‐Square Tests of Independence
Males	12.6	87.4	*χ* ^2^ (1) = 2.98
Females	19.9	80.1	*p* = .08
Non‐binary	11.1	88.9	n = 457
Sexing is fun and exciting			
Yes	68.3	68.3	*χ* ^2^ (1) = .006
No	31.7	31.7	*p* = .99
			n = 457
Sexting is safer than sex			
Yes	58.5	56.3	*χ* ^2^ (1) = .14
No	41.5	43.7	*p* = .71 n = 457
Sexting means you like the person			
Yes	50.0	58.7	*χ* ^2^ (1) = 2.06
No	50.0	41.3	*p* = .15 n = 457
Sexting should only happen between people in established relationships			
Yes	50.0	58.7	*χ* ^2^ (1) = 2.06
No	50.0	41.3	*p* = .15 n = 457
Sexting and sexual activity Which came 1st for you			
Sexted AFTER sexual activity	4.9	40.3	*χ* ^2^ (2) = 38.17
Sexted BEFORE sexual activity	87.8	53.6	*p <* .001
Sexted but not yet sexually active	7.3	6.1	V = 0.29*** n = 457

*p < .05. **p < .01. ***p < .001.

Next, we examined *risky* sexting‐related behavior, such as sending nudes in and outside a romantic relationship, body image, seeing a therapist, engaging in sexual activity in high school, and having participated in prevention programs such as school education about sexting. Having seen a therapist is a general indicator of emotional distress which is a broad and general indicator. However, our purpose was not to measure specific psychological difficulties but merely to separate those subjects who had experienced enough distress that they sought out professional help. We found differences between those who first sexted in middle school (early‐onset sexters; n = 82) and those who first sexted in high school (later adolescence sexters; n = 375) (Table 3). Non‐significant differences were found between early‐onset and later adolescence sexters in terms of sending nudes with romantic partners. However, significantly more early‐onset sexters (75.0%) than later‐onset sexters (66.4%) agreed with the statement “sexting makes me look critically at my body” (ϕ = .10; *p* = .04) Furthermore, early‐onset sexters were more likely (62.2%) than later‐onset sexters (40.8%) to have seen a therapist sometime during their high school career (ϕ = .17; *p* < .001). School‐based education on sexting had more impact on later adolescent sexters than early‐onset sexters (V = .14; *p* = .015): later onset sexters, if they were involved in sexting education at school (37.2%), were more likely to be impacted by the sexting education than early‐onset sexters (20.7%). When it comes to being sexually active with romantic partners v. non‐dates, early‐onset sexters showed a significantly higher sexual activity with non‐dates (23.2%) than later‐onset sexters (15.7%; V = .22; *p* < .001), the latter group being mostly active with romantic partners only (45.1%) (Table 3).

**Table 3:** Differences Between Early‐Onset (Middle School; MS) vs Later Adolescence (High School; HS) Sexters

Variable	MS (n = 82)	HS (n = 375)	Chi‐Square Tests of Independence
Send nudes more than one time per month to romantic partners			
Yes	35.4	26.9	*χ* ^2^ (1) = 2.35
No	64.6	73.1	*p* = n.s.
			n = 457
Send nudes more than one time per month to people other than romantic partners			
Yes	13.4	8.5	*χ* ^2^ (1) = 1.88
No	88.6	91.5	*p* = n.s. n = 457
Sexting makes me look critically at my body			
Yes	75.0	66.4	*χ* ^2^ (1) = 4.23
No	22.0	33.6	*p* = .04 ϕ= .10* n = 457
During high school, did you ever see a therapist?			
Yes	62.2	40.8	*χ* ^2^ (1) = 12.46
No	37.8	59.2	*p* < .001 ϕ= .17*** n = 457
Sexual activity in high school			
Sex w/romantic partner only	35.4	45.1	*χ* ^2^ (3) = 22.01
Sex w/non‐dates only	23.2	15.7	*p <* .001
Sex w/both romantic partner and non‐dates	24.3	8.8	V= .22***
Not sexually active	17.1	30.4	n = 457
School education about sexting			*χ* ^2^ (2) = 8.34
Yes, and it impacted me	20.7	37.2	*p* = .015
Yes, but it did not impact me	40.2	34.2	V = .14**
No	39.1	28.6	n = 457

*p < .05. **p < .01. ***p < .001.

Perceived pressure to sext differed by the age of first sext: those who started sexting in middle school felt significantly more pressure to sext than later adolescent sexters (V = .25; *p <* .001), and they were more likely than later adolescent sexters to have felt negative types of pressure, such as self‐pressure, being pestered, and very negative pressure (V = .20; *p =* .001). In contrast, friendly pressure was experienced at similar rates to both middle school and high school sexters (MS: 24.4%; HS: 24.8%). Although pressure to engage in sexual activity was perceived to be higher in later adolescence in general (MS: 13.4%; HS: 23.2%), early‐onset sexters felt both more pressure to sext, and an *intense* pressure to both sext and have sex, compared to later adolescence sexters (V = 0.17; *p <* .01). Early sexters were also significantly more concerned that they would be judged, called “frigid” or “slut” if they did not engage in sexting (ϕ = .18; *p <* .001) and they were more worried that others would not like them (ϕ = .22; *p <* .001). In the meantime, middle and high school sexters were equally likely to agree with the statement “too many people are pressured into sexting” (MS: 89.0%; HS: 86.9%; *p* = n.s.) (Table 4).

**Table 4:** Differences Between Early‐Onset (MS) vs Later Adolescence (HS) Pressure to Sext

Variable	MS (n = 82)	HS (n = 375)	Chi‐Square Tests of Independence
Too many people are pressured into sexting			
Yes	89.0	86.9	*χ* ^2^ (1) = .27
No	11.0	13.1	*p* = n.s.
			n = 457
Perceived pressure to sext			
No pressure	23.2	38.4	*χ* ^2^ (2) = 29.59
Some pressure	24.4	38.9	*p <* .001
Lots of pressure	52.4	22.7	V = .25***
			n = 457
Type of pressure felt to sext			
No pressure	15.9	37.6	*χ* ^2^ (4) = 18.61
Self‐pressure	19.5	14.9	*p =* .001
Friendly pressure	24.4	24.8	V = .20**
Pestered	31.7	17.1	n = 457
Very negative pressure	7.0	5.6	
Which pressure was more intense for me			
Pressure to sext	20.7	13.6	*χ* ^2^ (3) = 12.82
Pressure to have sex	13.4	23.2	*p <* .01
Both types of pressure equally intense	40.2	25.9	V = 0.17**
Neither type of pressure was intense	25.6	37.3	n = 457
Worried that others would judge me as frigid/slut if I did not sext			
Yes	56.1	33.9	*χ* ^2^ (1) = 14.14
No	43.9	66.1	*p <* .001
			ϕ = .18*** n = 457
Worried that others would not like me if I did not sext			
Yes	67.1	38.9	*χ* ^2^ (1) = 21.63
No	32.9	61.1	*p <* .001
			ϕ = .22*** n = 457

*p < .05. **p < .01. ***p < .001.

Two continuous variables measuring feelings of pressure to sext (represented on a Likert scale of 1 to 5 with lower numbers indicating less pressure) and how much the pressure bothered them (represented on a Likert scale of 1 to 4 with lower numbers indicating less burden) were both significantly different between the 2 groups. Early‐onset sexters reported feeling significantly more pressure to sext (*M* = 2.94, *SD* = 1.26) than did later adolescence sexters (*M* = 2.25, *SD* = 1.19), (*t*(455) = 4.70, *p* < .001, *d* = 1.20). In addition, those who started sexting in middle school felt significantly more bothered by the pressure (*M* = 2.67, *SD* = 1.17) than did later‐onset sexters (*M* = 2.09, *SD* = 1.14), (*t*(455) = 4.15, *p* < .001, *d* = 1.15) The effect sizes for these analyses (*d* = 1.20 and 1.15, respectively) were found to exceed Cohen's^48^ convention for a large effect (*d* = .80). (Table 5).

**Table 5:** Means and Standard Deviations for Early‐Onset (MS) vs Later Adolescence (HS) Pressure to Sext (*Lower Numbers on Scale Reflect Less Pressure/Burden*)

Variable	MS (n = 82), mean (SD)	HS (n = 375), mean (SD)	Cohen's *d*	Range
Did you feel pressure to sext?	2.94 (1.26)***	2.25 (1.94)***	1.20	1‐5
How much pressure did you feel to sext?	2.67 (1.17)***	2.09 (1.14)***	1.15	1‐4

*p < .05. **p < .01. ***p < .001.

## DISCUSSION

The research examined the patterns and poor health‐related consequences of pressured and coerced sexting comparing early‐onset (middle school) and late adolescence (high school) sexters on a cross‐sectional retrospective study on a large convenient sample of 3 public universities in the United States. Our data support previous research finding that more females are engaged in sexting than males.^16^, ^19^, ^22^, ^49^ Non‐binary adolescents were even more likely to engage in sexting in our sample. However, we cannot generalize these statements due to the low representation of non‐binary youth (n = 22). Most teenagers, independent of gender, started sexting between 16 and 17 years of age; however, female sexters were more active as early as 15 years old, compared to male and non‐binary sexters. This pattern can be explained by the early sexual maturation of females, relative to other sexes.^6^ In addition, our research shows that female sexting, even at an early age, often occurs in a relationship with a romantic partner. In contrast, more male and non‐binary sexting had to do with looking for a “hook up” or sexting with a friend instead of a romantic partner.

The data corroborate previous results on pressured sexting:^20^, ^22^ while most male adolescents experienced little pressure to sext, female and non‐binary sexters often felt some types of pressure, most likely friendly pressure (experienced by females) and being pestered (experienced by both female and non‐binary adolescents). It is worth mentioning that non‐binary adolescents felt the most “negative pressure,” while pestering and very severe pressure were almost non‐existent for males. Females personally experienced more pressure and were more likely to agree with the statement “females feel more expectations to sext,” compared to male and non‐binary individuals.

Normalization of sexting in adolescence was highly prevalent. Sexting as a normative behavior navigating everyday adolescent life was reflected in the statements “sexting is fun and exciting” and “sexting is safer than sex.” Most participants agreed with these statements, independently of whether they sent their first sext in high school or during middle school. Sexting often preceded sex for early‐onset sexters and was not necessarily connected to actual sexual conduct; however, later sexters often started sexual activity first, and they engaged in sexting as a part of their romantic relationships.

The study revealed unique patterns of early‐onset sexters compared to late adolescent sexters. Significantly more middle school than high school sexters believed that sexting was safer than sex, and more of them disagreed with the statement that one should like the person they sext with. Despite the relatively small proportion of early sexters in the sample (n = 82), the above beliefs indicate that sexting can be normative in early adolescence. Young adolescents who sext may find it safe to express sexual interest or perhaps simply to be entertained, even without sexual attraction. Clearly, sexting can happen at 15 years of age, or even earlier, and is not necessarily followed by actual sex; instead, it is part of regular, social communication.

Although negative health outcomes of sexting have been studied widely,^7^, ^14^, ^29^, ^31^, ^32^, ^33^, ^34^, ^35^, ^37^, ^50^ the prevalence and adverse psychological consequences of early sexting have been less researched.^6^ Our research provides a comparison of the magnitude and types of pressured feelings in both early and late adolescence. This study revealed that early‐onset sexters are at a higher risk of poor outcomes after sexting, compared to late adolescent sexters. Although there are certainly many factors that can influence teens' body image and seeing a school therapist as a consequence, the analysis shows strong statistical significance between early‐onset sexting and critical body views. Early‐onset sexters were also more likely than late adolescent sexters to see a psychologist/mental health professional during high school. In the meantime, early‐onset sexters were less likely to have been impacted by school‐based education on sexting than were older adolescent sexters. In addition, early‐onset sexters showed a significantly higher level of sexual activity with non‐dates than later‐onset sexters, who were sexually active, but mostly with romantic partners.

Our data revealed that early life sexting activity is more connected to feeling pressured than late adolescence sexting. Early‐onset sexters were more likely to feel pressured to sext and the pressure was more bothersome to them compared to later‐onset sexters. Thus, middle school sexters started sexting because they felt highly pressured to do so, by self‐pressure, persistent pressure (pestering), and adverse and aggressive pressure. Moreover, early‐onset sexters felt significantly more pressure to engage in sexting *and* have sex than later adolescent sexters; they were significantly more worried about being judged by peers as frigid or “slut,” or that peers will not like them if they do not engage in sexting.

## IMPLICATIONS FOR SCHOOL HEALTH

Utilizing a convenience sample, the current research cannot fully represent middle and high school population. Furthermore, self‐reporting of controversial or deviant adolescent behavior can be affected by information bias such as foggy memory^51^ and social desirability.^52^ Sensitive information (engaging in deviant behavior) may have therefore been underreported. Participation in the study was voluntary and confidential; however, it might be influenced by proposed incentives (course credit, gift card drawing) and a genuine interest in the field because of the participant's prior involvement in sexting. It is unsurprising that most participants were females, among whom sexting is more prevalent, who also experience more pressure to sext and who often start to engage both in sexting and sex earlier than males. Nevertheless, the sample provides an extensive and relatively equal representation of college students in 3 large public universities in the United States.

Due to the low number of gender minorities in our sample, we could not investigate the differences in pressured sexting for non‐binary youth. Although Van Ouytsel et al's^53^ study finds that gender minority students were more likely to have been pressured to send a sexting image than cisgender youth, research in non‐binary youth is scarce; this gap should be filled in the future. Researchers should explore the context of sexting, the mechanism and process of pressure, and the potential consequences of sexting on body image, self‐harm, social coping, and mental health issues. Disentangling the relationships among different types of sexting and health issues such as depression and self‐harm would also add to the development of evidence‐based recommendations. In addition, separating consensual from nonconsensual sexting is a critical next step for schools, law enforcement agencies, and policymakers. The current paper helps understand sexting vulnerability factors in teens, and provides valuable information to school administrations in teen sexting cases.

### Conclusions

Our study corroborated research findings by emphasizing the prevalence of sexting in females, non‐binary individuals, and high school students. It also contributed to existing research with new knowledge, highlighting that sexting can happen early, even in middle school. The underlying conditions of sexting for early‐onset (middle school) sexters include much more pressure, and more negative pressure, than later adolescent sexters. Our study also revealed that early sexting can have poor health‐related consequences, such as distorted body image and peer pressure‐related distress. Participants also revealed that school‐based sexting education is more impactful for older adolescents. Therefore, middle school adolescents may need age‐specific, and gender‐responsive sexting education that recognizes the role of peer pressure as a significant factor in sexting during early adolescence, and the gendered differences in pressured sexting. We suggest incorporating lessons/modules about sexting as well as sexuality‐related communications in sex ed. Finally, prevention and awareness‐raising programs about sexting should tackle digital health, safety, and security to help youth navigate their personal, social, and sexual development in a technological world. Age‐appropriate early‐life prevention and intervention programs should incorporate the psychological consequences of non‐consensual sexting.

### Human Subject Approval Statement

The research was reviewed by Virginia Tech, VA, IRB #20‐647 and Bridgewater State University, MA, IRB #2021001.

### Conflict of Interest

The authors declare that they have no known competing financial interests or personal relationships that could have appeared to influence the work reported in this paper.

## Data Availability

The data is available upon request.
